# 

**Published:** 1884-07

**Authors:** 


### BOOKS AND PAMPHLETS RECEIVED.

Studies from the Biological Laboratory, Johns Hopkins University. Edited by Newell M. Martin, M.D., and W. K. Brooks, Ph.D. Vol. 3, No. 1.

Annual Catalogue of the State Agricultural College, Wisconsin.

Bulletin of the American Museum of Natural History, Central Park, New York, Vol. 1, No. 5.

Report of the Commissioner of Agriculture, Washington, for the year 1883.

National Board of Health Documents, Washington, 1884.

List of Medical Works published by P. Blakiston, Son & Co., Philadelphia, during 1883-4.

American Veterinary College. Annual Announcement, Session 1884–5.

Johns Hopkins University Circulars.

Annals et Bulletin de la Sociétié de Medicine de Gand.

Journals.—La Clinica Veterinaria, Milano. The Veterinarian, London; The Veterinary Journal, London; Der Zoologische Garten, Frankfort; Schweizerisches Archiv fur Thierheilkunde, Zurich; Archiv fur Wissenschaftliche und Praktische Thierheilkunde, Berlin; Wochenschrift fur Thierheilkunde und Viehzucht, Augsburg; Journal de la Société contre L'Abus du Tabac, Paris; Tidskrift for Veterinar Medicine, Stockholm; Giornale di Anatomia, Fisiologia e Patologia, degli Animali, Pisa; Il Medico Veterinario, Torino; El Ensayo Medico, Caracas; La Cronica Medica, Valencia; Kansas City Review of Science and Industry; Chicago Medical Journal and Examiner; The College and Clinical Record, Philadelphia; New England Medical Monthly, Sandy Hook; Chicago Medical Review; Virginia Medical Monthly, Richmond; Nashville Journal of Medicine and Surgery; The Southern Clinic, Richmond; The Western Medical Reporter, Chicago; Journal of Cutaneous and Venereal Diseases, New York; Denver Medical Times; The St. Joseph Medical Herald; The Weekly Medical Review, St. Louis; The Blacksmith and Wheelwright, New York; National Live Stock Journal, Chicago; The Breeder's Gazette, Chicago; Cultivator and Country Gentleman, Albany; Weekly Drover's Journal, Chicago; The Chicago Tribune; United States Veterinary Journal, Chicago; Horseshoer and Hardware Journal, Chicago; The Polyclinic, Philadelphia; Medical World, Philadelphia; American Veterinary Review, New York; Atlantic Journal of Medicine, Richmond; San Francisco Western Lancet; Indiana Farmer, Indianapolis; Texas Courier Record, Fort Worth; American Psychological Journal, Philadelphia; The Jersey Bulletin, edited by D. H Jenkins, Indianapolis; American Journal of Ophthalmotology, edited by Dr. Adolf Alt, St. Louis, monthly, $2.50 a year; Pacific Medical and Surgical Journal, San Francisco.

